# Psychosocial Context of OSH-Remote Work of Academic Teachers in the Perspective of Sustainable Development

**DOI:** 10.3390/ijerph192214783

**Published:** 2022-11-10

**Authors:** Joanna Sadłowska-Wrzesińska, Kamila Piosik, Żaneta Nejman

**Affiliations:** 1 Institute of Safety and Quality Engineering, Faculty of Engineering Management, Poznan University of Technology, 2 Prof. Rychlewskiego Str., 60-965 Poznan, Poland; 2 OHS Sobczak—Training and Consulting Enterprise, 72 Bługarska STR., 60-321 Poznan, Poland

**Keywords:** work safety, COVID-19, stress, sustainability, teleworking, TOPSIS, AHP, occupational health

## Abstract

The purpose of the research paper is to analyse the factors affecting remote work in terms of the selected socio-economic criteria and to determine which elements contribute the most to the development of sustainable work. In addition, the study describes the issues of remote education at the academic level and the challenges faced by academic teachers during the COVID-19 pandemic. The whole is embedded in the issues of occupational health and safety, with particular emphasis on the psychosocial aspects of the occupational safety of academic teachers in Poland. In the research process, the TOPSIS multi-criteria analysis tool (technique for order of preference by similarity to ideal solution) was used, as well as AHP (analytical hierarchy process), as an auxiliary method. The use of these methods made it possible to select the most important variable and to determine the ranking of factors affecting the analysed problem. Findings: According to the conducted research, the most important factor affecting the safety of remote work—in relation to the selected sustainability criteria—is overwork/workload. An equally important element was stress during remote work, as well as the organization of time, with consideration to the balance between work and home duties. The research has shown that the selected aspects of remote work can have a significant impact on the achievement of sustainable development goals by a given organization, and in relation to individuals, on the quality of life and the sense of safety and health at work.

## 1. Introduction

Poland’s membership in the European Union leads to the obligation to adjust working conditions to the European standards. In the context of the organization of the labour protection system and the resulting guidelines, the emphasis has primarily been laid on minimizing risks and preventing psychosocial nuisances [1]. Moreover, shaping the optimal working conditions, in accordance with the principles of occupational health and safety, is one of the factors implemented as part of the sustainable development approach [2].

**Figure 1:**
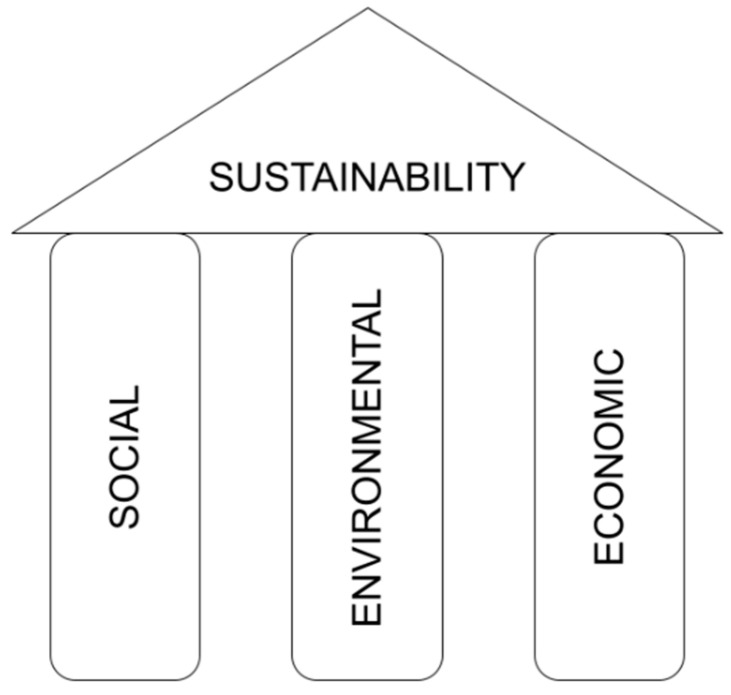
Model of three pillars of sustainable development. Source: own elaboration based on [3].

The most popular interpretation of the idea of sustainable development is the three-pillar model (Figure 1), illustrating three dimensions: environmental, economic and social resources. In this model, sustainable development is achieved when all three pillars work together, so consequentially the system is on the brink of a potential collapse if one of the pillars is unsustainable [3].

Being in balance has multiple meanings. The balance, usually described as the ability to self-regulate biological processes and as the ability to maintain the stability of certain life parameters, was inherently associated with the definition of health. Today, this concept has broader connotations. For example, attempts to solve current organizational problems are increasingly frequently often undertaken as a part of sustainable management, i.e., the process of achieving goals by the company in accordance with the principles of sustainable development.

By addressing the issues of sustainable management, the concept of sustainable work is defined as work that can be performed by the workers without prejudice to their physical and/or mental health, and which will take place for an extended period of their professional occupation [4]. Eurofound specified the definition of “sustainable work throughout life” and placed emphasis on the fact that working and living conditions encourage people to take up and keep a job through an extended professional occupation. These conditions make it possible to tailor an occupation to the characteristics of a person and can be developed by changing the life of a person, by the means of rules and practices at work and outside the work [5]. The sustainable development term was first referred to the occupation by Scandinavian researchers who contrasted sustainable work systems with intensive work systems. The authors argue that the latter labour systems have a detrimental effect not only on individuals but also on the quality of products and services, in long term. They proposed the organization of work in such a way that it is possible to “regenerate human and social resources” [6].

The events caused by the outbreak of the COVID-19 pandemic initiated changes in all areas of human life, from the changes in the global economy, through health protection and modification of interpersonal relations, to professional work, its new forms, time, scope and organization [7,8,9,10].

Many enterprises and practically all educational and scientific units have introduced a remote work model, which has been associated with the transformation of work conditions and organization of work, as well as a significant increase in mental workload. Already before the pandemic, mental health problems affected about 84 million people in the EU [11]. Half of European workers believe that stress is common in their workplace and that it contributes to around half of all lost working days. Nearly 80% of managers are concerned about work-related stress [12], bearing in mind its negative effects on the well-being of employees and, as a result, on the well-being of the entire organization.

As a result of the pandemic, nearly 40% of employees started working remotely in full-time extent. These figures are impressive when compared to the beginning of 2020, when only one in ten people employed in the EU worked remotely, in full-time or part-time, in the professions requiring high skills, such as information and communication technology (ICT) sectors [13]. The current situation removes traditional boundaries between work and private life, and together with other trends in remote work, such as constant connectivity, lack of social interaction and increased use of ICT, gives rise to psychosocial and ergonomic threats. There is an increasing number of projects that aim to develop and implement interventions for the promotion of good mental health and prevention of mental illness at work [14]. However, they mainly concern healthcare workers.

The specificity of an academic teacher’s work cannot be put into rigid frames and simply described, although it definitely belongs to socially important professions. The polysemantic nature of the academic teacher profession and multitasking are inherent in this profession. The academic teacher’s work, primarily a didactic and scientific work, is a creative, time-consuming and responsible occupation. The importance and multidimensionality of this work should be emphasized, as well as the systematically increasing scope of administrative and supervisory duties not directly related to teaching. These conditions contribute to the growing frustration in this professional group. Nearly 100,000 academic teachers are employed in Poland (data from 2021), of which approx. 90% in public universities [15]. University research and teaching staff, according to research conducted by CPOR (The Center for Public Opinion Research is a centre that conducts surveys representative of the Polish society), is respected by 83% of the society [16]. During the pandemic, academic teachers faced a significant challenge to reorganize the entire teaching process, and its course was often dependent on the level of digital competences, as well as personal commitment and creativity [17]. Moreover, the key task was to prepare content, teaching materials and teaching aids, that are attractive from the perspective of the recipients, i.e., students. Nevertheless, the real challenge was to motivate students for independent searching and research work. This task was perhaps the most challenging, because online learning tools are imperfect and in most cases do not allow for the full achievement of educational goals. However, the positive aspects of the forced reorganization of the teaching process should be emphasized—observations by Sjølie, El and others demonstrated that the time of the pandemic has renewed the community character of academic life. At the time of remote work, we appreciated academic life and work much more, we also regained what we value most in academic life and work: its internally communal character [18,19,20,21].

The negative aspects of the forced reorganization of the teaching process primarily included new type of barriers, such as protective masks, visors, social distance, restricted contact, etc. These barriers, although necessary to minimize the risk of infection during the COVID-19 pandemic, negatively affected the chances for the selection of teaching methods, student-teacher relationship and, moreover, limited the variety and richness of educational exchanges [22,23,24]. The negative aspects of remote work in a non-teaching context should also be mentioned. Information chaos, disturbed decision-making process and the dynamics of changes regarding the closing and reopening of schools all had a significant share in the teachers’ experience of anxiety and fear. Moreover, some teachers were more likely to leave the profession [25]. Another example can be provided by the results of research by Allen, Jerrim and Sims, which demonstrated that the pandemic evidently had a negative impact on the mental health of teachers [26].

Working at a university is a special type of professional activity. The multidimensionality means that some of its aspects escape definitions. According to the authors, this is another argument for further research conducted on this professional group. In the literature of the subject, one can find studies on sustainable education [27,28] as well as those concerning the sustainable development of digitization in higher education [29,30]. However, there is a clear lack of studies on the sustainable work of academic teachers. The authors decided to fill this research gap.

## 2. Materials and Methods

The aim of the research was to identify the key variables that are most important for sustainable work. To achieve this goal, the TOPSIS multi-criteria analysis was carried out and auxiliary methods were used, such as: questionnaire, MICMAC and AHP. In the first stage of research [31], a literature analysis was conducted and on its basis the researchers determined 20 factors affecting remote work. Then, this number was limited based on the results of a survey conducted among employees of the Faculty of Management Engineering at the Poznań University of Technology (*n* = 50). The survey was conducted during the second and third waves of the pandemic in Poland. The questionnaire consisted of 20 questions which the respondents had to answer by selecting one of the five possible variants. The questions were developed on the basis of factors selected as a result of a literature review in the field of remote work safety. Among them, the following were distinguished: professional communication with colleagues, organization of working time, stress, time pressure, motivation, job satisfaction and adaptation to new technologies [31]. The next research step was the structural analysis with the use of MICMAC software. MICMAC method (matrix-based multiplication applied to a classification) is applied for testing structural factors by using a study of relations between them and their importance in the system. Thanks to MICMAC analysis it is possible to explain how data will function and in which way they should be managed. This technique uses a matrix containing all possible pair of factors, in which can be found relation or not (A factor → B factor). What is important, MICMAC program gives an opportunity to show results in graphic way, thanks to impacts graphs and also relations and impacts maps [31]. The graphs show correlations between data, taking into consideration their intensity. There are five kinds of relation strength: the weakest, weak, medium, relatively strong and strong. In our research the following factors are selected: technical supplies, a sense of independence, workload, organization of time between work and home duties, work outside the set hours, time pressure, professional communication with colleagues, and stress [31].

The AHP method was used to calculate the weights of selected criteria. Both AHP and the TOPSIS analysis used for decision making are presented in this study.

The AHP (analytical hierarchy process) method, introduced in 1980 by Thomas Saaty, assumes the breakdown of a particular problem into its components and the creation of a hierarchy for the resulting set of alternatives [32]. AHP is one of the best known and most frequently used MCA (multicriteria analysis) approaches. It is based on five steps that show how to proceed during the analysis. The AHP method has been used in many different fields over past 40 years. Due to the possibility of multi-criteria analysis of complex decision problems, researchers used it in a variety of ways, for example:

- To determine the relative weights of the elements used in the target programming model for the selection of IS [33];
- To select a multimedia authorial system on the basis of a case study [34];
- To choose an ERP system, i.e., software for comprehensive enterprise management [35];
- To examine its impact on project management, in particular the selection of subcontractors [36];
- To select a telecommunications system supplier on the basis of a case study [37];
- To analyse the choice of a transhipment port by global carriers [38];
- To choose the location of the facility from the point of view of organizations that are considering introducing a new branch or relocating existing branches [39].

The main purpose of using AHP in our research was to find the weight of social-economics criteria in the area of sustainable development. In order to determine them, there was a need to conduct five stages of the method: stage I—the identification of problem and the definition of goal; stage II—the elaboration of hierarchy (starting from the most important aspects of decision-maker opinion, then intermediate level, i.e., elements on which next criteria depend, and finally the least important alternatives according to decision-maker’s mind); stage III—the construction of matrix comparing pairs of alternatives (each criterion is compared to others, using 10-points scale); stage IV—the hierarchical synthesis; stage V—the examination of the consistency of matrix by calculating λ_max_, CI, RI, CR indicators.

TOPSIS (technique for order of preference by similarity to ideal solution) is a multi-criteria method used in making decisions. It was introduced for the first time by Hwang and Yoon in 1981, and its main idea is derived from the concept of seeking an ideal and anti-ideal solution. This method is based on the assumption that the most appropriate alternative has not only the shortest distance from the best solution, but also the longest distance from the worst solution [40]. TOPSIS helps decision makers solve problems through analysis, comparisons and ranking the alternatives, so it can be used in many different fields.

## 3. Results

**Figure 2:**
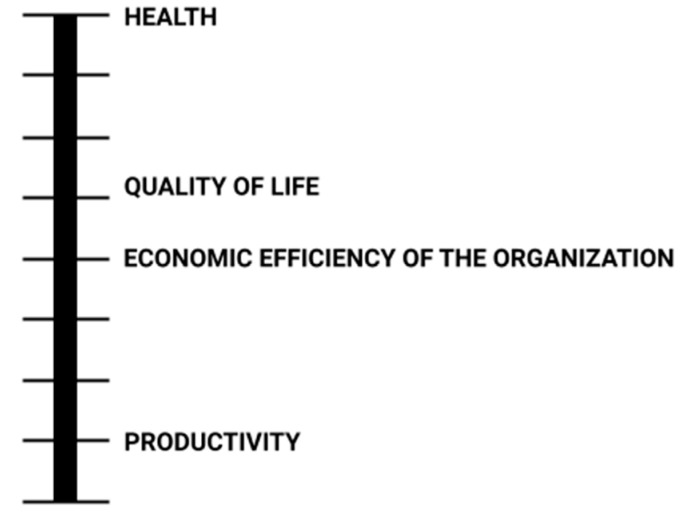
Hierarchy of criteria. Source: own elaboration.

Thanks to the application of the AHP method, the weights of socio-economic criteria in the field of sustainable development have been established. In order to determine them, it was necessary to carry out five steps of the above method. First of all, based on the literature review, the authors identified the criteria relating to sustainable development [31], and then hierarchy of these criteria was developed (Figure 2) The health and safety of the employee was considered the most important aspect, and the quality of life was second. The economic efficiency of the organization was classified immediately behind it. The productivity was considered the least significant among the selected categories.

In the next step, the criteria were compared in pairs to establish their precedence or subordination to each other. Each of the elements was compared to the rest of them, using the scale below (Table 1).

At this stage of the research, a comparative matrix was constructed (Table 2). It showed the relations between the criteria, with means of numerical values.

The next step was focused on performing a hierarchical synthesis, consisting in dividing each matrix element by the sum of its column (Table 3).

The averages in each row were then calculated (Table 4). Their sum should be approximately equal to 1. Based on the results obtained, the so-called priority vectors were obtained, i.e., the weights of the given criteria.

Then the means for each row were calculated (Table 4). Their sum should be approximately equal to 1. Based on the obtained results, the so-called priority vectors, i.e., weights of given criteria, were obtained. In the last stage of the AHP method, consistency of the matrix was tested. In order to assess whether the values (Table 2) were given correctly, whether the *CR* index was lower than 0.1. was checked For this purpose, the *λmax*, *CI*, *RI* indices were calculated. We started by adding up the products of the priority vectors and the values in the columns of the comparative matrix:

(1) $$0.597\left[\begin{array}{c} 1\\ 1/4\\ 1/8\\ 1/5 \end{array}\right]+0.214\left[\begin{array}{c} 4\\ 1\\ 1/5\\ 1/2 \end{array}\right]+0.05\left[\begin{array}{c} 8\\ 5\\ 1\\ 4 \end{array}\right]+0.139\left[\begin{array}{c} 5\\ 2\\ 1/4\\ 1 \end{array}\right]=\left[\begin{array}{c} 2.549\\ 0.892\\ 0.202\\ 0.566 \end{array}\right]$$

The obtained values were divided by the corresponding priority vectors:

(2) $$\frac{2.549}{0.597}=4.272\;\frac{0.892}{0.214}=4.177\;\frac{0.202}{0.05}=4.039\;\frac{0.566}{0.139}=4.056$$

Then their average was calculated, and the *λ_max_* index was obtained.

(3) $$\lambda_{max}=\frac{\left(4.272+4.177+4.039+4.056\right)}{4}=4.136$$

In the next step, the consistency index (*CI*) was determined.

(4) $$CI=\frac{\lambda_{max}-n}{n-1}=\frac{4.136-4}{4-1}=0.045$$

The obtained consistency index was divided by the value of the *RI* coefficient, which depends on the size of the matrix and results from the data presented in Table 5.

On this basis, the coefficient of consistency of the matrix was determined.

(5) $$CR=\frac{CI}{RI}=\frac{0.045}{0.9}=0.05\;CR<0.1$$

The calculated *CR* coefficient is less than 0.1, which means that the relations between the criteria were correctly assessed.

The TOPSIS analysis started with the design of a matrix including the economic and social criteria and factors related to remote work. The selection of these elements results from the earlier stages of the research process. The individual values in the decision matrix were determined by a group of decision makers. It was composed of the Dean of the Faculty of Management Engineering, Vice-Dean for Science, Vice-Dean for Education and Student Affairs, and Vice-Dean for International Relations and Business. Then the arithmetic mean of the grades was determined for the Authorities of the Faculty of Management Engineering at the Poznań University of Technology. The averaged matrix is presented in Table 6.

In the next step, the average decision matrix was normalized. Thanks to these transformations, a dimensionless matrix was obtained. Then, the values from the normalized matrix were multiplied by the criteria weights obtained on the basis of the AHP analysis (Table 7).

At this stage of the research, the values of A+ and A− were determined; they are the so-called ideal and anti-ideal solutions (Table 8). The first ones take into account the smallest values.

In the next step, the distance of the tested variants from the ideal and anti-ideal solutions was calculated (Table 9). This distance is called the Euclidean distance.

Subsequently, a ranking coefficient was determined for each of the examined factors (Table 10). Based on its value, a ranking was developed in which the variant with the highest index is in the highest position, and thus it can be considered the most important.

The analysis of the obtained indices showed that the highest value was achieved by the factor “Overwork/workload”, for which Ri is equal to 0.9900. This factor achieved the first position in the ranking, therefore it can be considered the most important. The next factors that obtained significant results in the ranking are as follows: “Stress” (Ri = 0.9899), “Time organization between work and household duties” (Ri = 0.8483) and “Time pressure” (Ri = 0.6343).

## 4. Discussion

### 4.1. Sustainable Development of Academic Teachers while Working Remotely

Remote education is demanding and requires self-discipline and regularity, as well as conditions for which neither students nor academic teachers were prepared. With regards to the students, even the efficient use of modern information technologies does not mean the ability to organize your own learning process. With regard to teachers, e-learning requires (apart from digital competences) more dedicated time than real-life education and classroom education.

Research conducted in Poland [42] showed that at least four out of five academic teachers had no contact with remote learning before the pandemic. The outbreak of the pandemic meant that 80% of academic teachers had to completely change the methods of education as a result of a single ordinance of the rectors, in a single day. From the perspective of the two-year-plus period of the pandemic, it should be stated that we were not prepared for it—not from the formal and legal point of view, or from the infrastructural point of view (the IT infrastructure did not include the rapid increase in users over several days), or methodically. This state of affairs is also confirmed by research in other countries [43,44]. Despite the use of many technological solutions, the period of the pandemic illustrated the scale of previously underestimated educational problems. Shifting the university’s everyday life into the virtual world, in order to maintain the continuity of the academic education, was a duty of academic teachers. These are several problematic areas that require specific and continuous attention: the digital competences of research and teaching staff and the technical infrastructure of the university (equipment and internet connections). The remaining problems seem to be universal for all working people—during subsequent lockdowns and the need to switch to home office model, we had to deal with the organization of working time, find the balance between home and work duties, and provide childcare. In the course of remote work, everyone was additionally accompanied by fear resulting from the lack of control and fear related to the lack of appropriate knowledge and digital competences.

Digital competences are included in the eight key competences in the lifelong learning process [45]. The DESI report shows that in 2017, 44% of the population of the European Union had low digital skills or no digital skills at all (19%). Interestingly, over the last four years, this level has increased to 58% in the area of basic digital skills. A total of 82% of people aged 16–24, 68% of employees or self-employed, and 87% of students have at least basic digital skills. However, in the group of 55–74-year-old and retired people, it amounts to 35% and 30%, respectively [46]. Digital economy is also affected by the so-called digital exclusion. This phenomenon consists of not only the lack of access to the Internet (its quality, speed and stability of the link), but also the availability of hardware and software, its quality and the ability to use it (e.g., laptop, tablet, smartphone or scanner). After the lockdown was announced, some students left for their hometowns (often small towns and villages) without broadband Internet or an extensive technical background (printers, cameras, speakers, headphones, microphones or necessary applications or computer programs). Technological barriers on the side of the recipient (students, pupils) disrupted the education process and had an impact on the comfort of teachers’ work.

Our research confirmed that teleworking is only seemingly common and easy, which is also emphasized by other authors [47,48]. We have tried to show that the COVID-19 pandemic has severely limited a teacher’s ability to maintain high-quality education. Restoring the quality of teaching and learning is essential for the proper achievement of sustainable development goals and strengthening teachers’ leadership [32]. Scientists, decision makers in education and the public agree that teachers should be seen as very joyful and thus ‘infect’ their students with the excitement of learning. However, the problems arising from the COVID-19 pandemic have strengthened fear and anxiety [49], increased the level of stress [50], and induced physical health problems (e.g., pain/weakness, weight gain) [51]. The above-mentioned factors significantly limit the sustainable development of academic teachers in the work environment.

### 4.2. Remote Work and Occupational Health and Safety

Our research results show that the most important factor affecting remote work (in relation to the selected sustainability criteria) is overwork/workload. At the same time, there is an opinion among employers that teleworking is associated with an increase in productivity. In a specific unit of time, an employee can do more work and achieve better results. Unfortunately, observations of the working environment suggest that the “productivity improvement” may be due to fewer breaks and also due to the flexibility of some employees when scheduling their own work. The result of such an approach is “non-stop” working and being in constant readiness. Some authors confirmed in their studies that seemingly better productivity may be dictated by longer working hours and the intensity of the tasks performed. An extremely interesting study by Montreuil and Lippel [52]. In six companies in Quebec, Canada, demonstrated that nearly all of the teleworkers and their supervisors under study reported that their productivity increase resulted from fewer brakes. Many of them mentioned more working hours in the first weeks of this form of work, while some of them emphasized that a year after starting teleworking, they continued to work longer than required. In another company surveyed, it was stated that longer working hours have become an integral part of the company’s organizational culture.

Another factor motivating employees to operate more efficiently may be the willingness to prove the effectiveness of such an alternative form of work as well as their own usefulness in the organization. The feeling of uncertainty and fear of securing one’s livelihood are not surprising when the data collected by the University of Warsaw researchers are taken into account. After the first year of the pandemic, approx. 660 thousand people lost their jobs in Poland. Household incomes also decreased: as many as 30% declared a lower income [53].

Mental and physical comfort while performing work from home is an important aspect that may affect the employee’s health. This is confirmed by the research conducted by the authors, which shows that overwork, stress and the problem of work organization, with consideration to the division into work and home duties, are the most important factors affecting the safety of remote work. It is generally recommended to plan your own work area in a room separate from the rest of the house, if it possible. A dedicated office can help identify the physical barrier between private and professional life. People participating in the Montreuil and Lippel study [54] confirm that it is important to have a separate workstation and to secure a time gap with a calm atmosphere at home. They also indicated that they generally prefer the home environment, because of its quiet nature, better air quality and temperature control, which affects their concentration. The importance of safe and hygienic working conditions and an appropriately adapted workstation should be an aspect of particular importance for the employer when organizing remote work. Similarly to shopping for a traditional office, employees should obtain the necessary equipment and materials or at least participate in the selection of equipment through advice in terms of ergonomics and health and safety. However, such an approach requires appropriate legal regulations in the field of labour protection. A separate problem is the issue of organizing one’s own time, and especially the performance of professional duties in hours other than customary. Earlier studies have clearly shown a significant impact of such work on both health and the subjective well-being of employees [55].

To perform the professional duties as a remote work requires to use of a computer. It involves frequently adapted wrong body posture, including the back, forearms, and wrists, as well as repetitive movements, as well as long, uninterrupted periods of continuous work. These elements may contribute to the development of musculoskeletal problems, with particular emphasis on the lumbar region, hands, wrist, arms and also the neck [52,56]. One of the possibilities of solving these problems and reducing their negative effects is the use of ergonomic equipment, characterized by, inter alia, the possibility of adapting a given component to the individual characteristics of the user. However, the appropriate infrastructure is not always sufficient to ensure safe work. The employees should have a basic understanding of ergonomics, which will help to avoid unhealthy postures. The amount of time spent working at the computer is also worth noting. When it is too long, it can cause, inter alia, pain in the spine, eyes and head. Therefore, it is important to spend time outside work in an active way, e.g., to go for a short walk. Standard office work gives you the opportunity to take breaks that are beneficial for your health, for example for social meetings. What is important is that a remote worker should employ some self-discipline to take the necessary breaks and only work at the computer for a specified period of time [56,57].

In the special case of telework, isolation should also be considered, which is regarded as one of the main disadvantages of this form of work. A certain type of exclusion from social life in the scope of the professional environment may lead not only to a sense of loneliness, but also to the lack of or lesser participation in special projects and professional activities, with the resulting limited attention from the superiors and reduced possibility of promotion. In addition, the state described may raise concerns about being forgotten by people in the office [25,50]. Another issue contributing to the development of work-related mental health problems may be difficulties in keeping the boundaries between work and home and family. It may lead to increased stress and even professional burnout [51]. Another reason for excessive stress may be the need to deal with technical problems on your own. Provision of support by the employer and superiors in the form of training and direct assistance regarding the operation of equipment, computer programs, communication systems, etc., may be very helpful at time of remote work. Our research has not confirmed that technical problems are an important safety factor when working remotely taking into account the aspect of sustainable development; however, this may result from the individual vetting of a given organization to work remotely.

The economic aspects as well as increased flexibility for both employees and the organization are undeniable advantages. However, new problems related to the impact of this form of work on the health and safety of employees arise. New threats may affect both the physical and mental safety of a person, therefore it is particularly important to recognize and minimize them. In our research, we take up an important aspect of the contemporary work environment and try to find an appropriate methodology for diagnosing current problems.

### 4.3. Limitations and Perspectives

In this study, an attempt was made to identify variables affecting the remote work and to indicate the most important of them from the perspective of sustainable development assumptions. The survey results show the elements of remote work, on which an organization should focus its efforts, in order to secure the highest possible level of comfort for its own employees, with consideration to various aspects of psychosocial safety. The findings of the research presented in this study and its considerations do not exhaust all problems related to the safety of remote work. On the contrary, they indicate the direction and opportunities for research improvement in this area, and may also prove helpful in the operational activities of an organization focused on sustainable development.

The authors are aware of the limitations of their own research, but also the strengths of the study. One of the strongest points is the research methodology, which significantly differs from surveys that are prevailing in this area. A different methodological approach allowed to look at the problem from a slightly different perspective, and the multi-criteria analysis tools used in the study allowed for the objective valuation of previously obtained opinions. According to the authors, it is an interesting and intellectually attractive procedure. A weakness of the survey is its focus on a selected (unrepresentative) group of academic teachers, which means that this study is an undertaking strictly related to its application in the context of a given organization, and it lacks universality. However, the authors hope that the presented methodological approach will interest other researchers in the area of occupational health and safety. The sense of safety is always multidimensional; therefore its measurement should be based on multiple criteria. Unfortunately, practice shows that, most often, measurement is based on survey research (a checklist), that is based solely on self-assessment or expressing opinions/beliefs on a given topic. The use of multi-criteria analysis methods contributes to better forecasting of future events, their prevention and preparation for their occurrence. It improves our sense of safety and thus the quality of life.

Obviously, the authors are aware that in spite of the efforts towards the maximum objectivity of the results, it is impossible to avoid some level of subjectivity. In multicriterial analysis there is no theoretical basics for constructing hierarchy, which affects the subjectivity of the final rankings (AHP); the subjectivity is visible also during creation of decision-making matrix (TOPSIS). Moreover, the methods are quite fragile because of basing on arbitrary scale of grades—the value data are defined by the decision-maker in verbal way, so they can change depending on the tested object and harvest, where this object is located. Among the advantages of the mentioned methods there are the possibility to use both the measurable and non-measurable elements in one model, the possibility to classify and organize analysed decision problems, the opportunity for fast identification of the best possibility and also the control of rationality during the study.

In the scope of further research, the authors wish to focus on the positive aspects of the changes implemented by the COVID 19 pandemic in the working environment of academic teachers. As an example, the DESI report shows that there has been a significant increase in digitization compared to the period before the pandemic in all member states, although the leaders of digitization in Europe are still Finland, Sweden and Denmark [46]. The authors, in the near future, would like to continue research on the psychosocial aspects of occupational safety in the group of academic teachers, with the wish to place such research in the area of positive psychology.

## 5. Conclusions

The subject of this study was the examination of factors affecting the safety of remote work, with consideration to the aspect of sustainable development. The aim of the study was to identify variables affecting remote work and to indicate the most important of them from the perspective of selected socio-economic criteria. In the research process, the TOPSIS multi-criteria analysis tool and the auxiliary AHP method were used. The use of these methods made it possible to select the most important variable and to determine the ranking of factors influencing the analysed problem.

The material collected in the course of the research and the performed analysis show that the most important factor affecting remote work (in relation to the selected sustainability criteria) is overwork/workload. The reason is probably the excess of obligations resulting from different nature of work, too many tasks to be performed in a short time, but also, the lack of standardized rules related to the new approach.

The above-mentioned elements are directly related to the next factor in the ranking, which was stress during remote work. The new social reality caused by the pandemic, naturally occurring problems and difficult situations induce tension and contribute to the feeling of uncertainty and fear.

Time pressure and working outside the designated hours, according to the research results, should be treated as other factors affecting the safety of remote work. For certain teachers, these factors may be perceived as problematic and lead to negative results in terms of well-being and satisfaction from the performed job. For others, these factors are not important, which can be explained by routine, as well as the fact that many academic teachers are accustomed to working under time pressure and at non-standard hours.

An interesting aspect of the research is its result indicating the relatively low importance of technical equipment as a factor affecting the safety of remote work, with consideration to the aspect of sustainable development. It is quite similar in the case of professional communication. The minor importance of this factor may result from the use of appropriate communication channels and knowledge of internal procedures in this scope of the university’s activity.

According to the results of the research, the sense of independence is the least significant factor affecting the safety of remote work, with consideration to the aspect of sustainable development. The form of work (stationary vs. remote) does not have a substantial impact on the autonomy and freedom in performing professional duties in the group of academic teachers.

The concept of sustainable development is inextricably linked with the knowledge of human well-being and the ways of achieving it. Among the latest scientific studies, there are a number of studies related to sustainable teaching or the sustainable development of digitization in higher education, while there is no research on the sustainable work of academic teachers. In particular, it should be emphasized that there are no studies showing a link between sustainable development and aspects of occupational safety and health.

The applied methods of multi-criteria analysis allowed for the objectification of the opinions of respondents (obtained in the previous survey) on the factors influencing remote work. Taking into account that the commonly used research approach in the area of psychosocial aspects of OSH is measurement based on respondents’ self-esteem or expert assessment (a checklist), the use of multi-criteria analysis is an intellectually attractive procedure and inspiring for further research. The authors are convinced that the presented research methodology may be useful in terms of application to the operational activities of organizations focused on sustainable development.

## Figures and Tables

**Table 1:** Values that define the relationship between criteria.

If the Fulfilment of the i-th Criterion Takes Precedence or is Higher in the Priority Hierarchy	If the Fulfilment of the i-th Criterion Has No Precedence or Is Lower in the Priority Hierarchy
1 = no priority	1 = no priority
2 = between 1 and 3	1/2
3 = slight priority	1/3 = slight subordination
4 = between 3 and 5	1/4
5 = clear pronity	1/5 = clear subordination
6 = between 5 and 7	1/6
7 = very clear priority	1/7 = very clear subordination
8 = between 7 and 9	1/8
9 = indisputable priority	1/9 = indisputable subordination

Source: [41].

**Table 2:** Comparison matrix.

	Employee Health and Safety	Quality of Life	Productivity	Economic Efficiency of the Organization
Employee health and safety	1/1	4	8	5
Quality of life	1/4	1/1	5	2
Productivity	1/8	1/5	1/1	1/4
Economic efficiency of the organization	1/5	1/2	4	1/1
Sum	1.575	5.7	18	8.25

Source: own elaboration.

**Table 3:** Hierarchical synthesis.

	Employee Health and Safety	Quality of Life	Productivity	Economic Efficiency of the Organization
Employee health and safety	1/1.575	4/5.7	8/18	5/8.25
Quality of life	0.25/1.575	1/5.7	5/18	2/8.25
Productivity	0.125/1.575	0.2/5.7	1/18	0.25/8.25
Economic efficiency of the organization	0.2/1.575	0.5/5.7	4/18	1/8.25

Source: own elaboration.

**Table 4:** Hierarchical synthesis with arithmetic averages included.

	Employee Health and Safety	Quality of Life	Productivity	Economic Efficiency of the Organization	The Arithmetic Mean of the Row
Employee health and safety	0.635	0.702	0.444	0.606	H_1_ = 0.597
Quality of life	0.159	0.175	0.278	0.242	H_2_ = 0.214
Productivity	0.079	0.035	0.056	0.030	H_3_ = 0.050
Economic efficiency of the organization	0.127	0.088	0.222	0.121	H_4_ = 0.139
		SUM			1

Source: own elaboration.

**Table 5:** Indicator values RI.

Matrix Size	1	2	3	4	5	6	7	8	9	10
RI	0	0	0.58	0.9	1.12	1.24	1.32	1.41	1.45	1.49

Source: [36].

**Table 6:** Average decision matrix for the Authorities of the Faculty of Management Engineering.

Criterion Factor	Employee Health and Safety	Quality of Life	Productivity	Economic Efficiency of the Organization
Professional communication with colleagues	3	3.25	4.5	4
Organization of time between work and household duties	4.25	4.5	3.75	4.25
Working outside of designated hours	3.75	4.5	3.5	3.75
Overwork/workload	4.75	5	3.75	4.25
Stress	4.75	5	3.75	4
Time pressure	4	4.5	3.25	4
Sense of independence	3	3.75	3	3.25
Technical supplies	3.5	3.25	4	3.75

Source: own elaboration.

**Table 7:** Matrix normalized with criteria weights.

Criterion Factor	Employee Health and Safety	Quality of Life	Productivity	Economic Efficiency of the Organization
Weights	0.597	0.214	0.139	0.050
Professional communication with colleagues	0.161	0.058	0.060	0.018
Organization of time between work and household duties	0.228	0.080	0.050	0.019
Working outside of designated hours	0.201	0.080	0.046	0.017
Overwork/workload	0.255	0.089	0.050	0.019
Stress	0.255	0.089	0.050	0.018
Time pressure	0.215	0.080	0.043	0.018
Sense of independence	0.161	0.066	0.040	0.015
Technical supplies	0.188	0.058	0.053	0.017

Source: own elaboration.

**Table 8:** Determination of the ideal and anti-ideal solution.

Criterion Factor	Employee Health and Safety	Quality of Life	Productivity	Economic Efficiency of the Organization
Weights	0.597	0.214	0.139	0.050
Professional communication with colleagues	0.161	0.058	0.060	0.018
Organization of time between work and household duties	0.228	0.080	0.050	0.019
Working outside of designated hours	0.201	0.080	0.046	0.017
Overwork/workload	0.255	0.089	0.050	0.019
Stress	0.255	0.089	0.050	0.018
Time pressure	0.215	0.080	0.043	0.018
Sense of independence	0.161	0.066	0.040	0.015
Technical supplies	0.188	0.058	0.053	0.017

Source: own elaboration.

**Table 9:** Calculation of Euclidean distance.

Factor	di+	di−	di+ + di−
Professional communication with colleagues	0.0098	0.0004	0.0102
Organization of time between work and household duties	0.0009	0.0051	0.0060
Working outside of designated hours	0.0032	0.0021	0.0053
Overwork/workload	0.0001	0.0099	0.0100
Stress	0.0001	0.0099	0.0100
Time pressure	0.0020	0.0034	0.0054
Sense of independence	0.0098	0.0001	0.0098
Technical supplies	0.0055	0.0009	0.0064

Source: own elaboration.

**Table 10:** Determination of the ranking factor.

Factor	Ri	Ranking
Professional communication with colleagues	0.0401	7
Organization of time between work and household duties	0.8483	3
Working outside of designated hours	0.3992	5
Overwork/workload	0.9900	1
Stress	0.9899	2
Time pressure	0.6343	4
Sense of independence	0.0065	8
Technical supplies	0.1408	6

Source: own elaboration.

## Data Availability

Not applicable.
